# Mechanisms of Quercetin against atrial fibrillation explored by network pharmacology combined with molecular docking and experimental validation

**DOI:** 10.1038/s41598-022-13911-w

**Published:** 2022-06-13

**Authors:** Xin Tan, Wei Xian, Xiaorong Li, Yongfeng Chen, Jiayi Geng, Qiyi Wang, Qin Gao, Bi Tang, Hongju Wang, Pinfang Kang

**Affiliations:** 1 Department of Cardiovascular Disease, the First Affiliated Hospital of Bengbu Medical College, 287 Chang Huai Road, Bengbu, Anhui 233004 People’s Republic of China; 2 Department of Psychiatry, Bengbu Medical College, Bengbu, People’s Republic of China; 3 Department of Physiology, Bengbu Medical College, Bengbu, People’s Republic of China; 4 Key Laboratory of Basic and Clinical Cardiovascular and Cerebrovascular Diseases, Bengbu Medical College, Bengbu, People’s Republic of China

**Keywords:** Cardiovascular diseases, Arrhythmias

## Abstract

Atrial fibrillation (AF) is a common atrial arrhythmia for which there is no specific therapeutic drug. Quercetin (Que) has been used to treat cardiovascular diseases such as arrhythmias. In this study, we explored the mechanism of action of Que in AF using network pharmacology and molecular docking. The chemical structure of Que was obtained from Pubchem. TCMSP, Swiss Target Prediction, Drugbank, STITCH, Pharmmapper, CTD, GeneCards, DISGENET and TTD were used to obtain drug component targets and AF-related genes, and extract AF and normal tissue by GEO database differentially expressed genes by GEO database. The top targets were IL6, VEGFA, JUN, MMP9 and EGFR, and Que for AF treatment might involve the role of AGE-RAGE signaling pathway in diabetic complications, MAPK signaling pathway and IL-17 signaling pathway. Molecular docking showed that Que binds strongly to key targets and is differentially expressed in AF. In vivo results showed that Que significantly reduced the duration of AF fibrillation and improved atrial remodeling, reduced p-MAPK protein expression, and inhibited the progression of AF. Combining network pharmacology and molecular docking approaches with in vivo studies advance our understanding of the intensive mechanisms of Quercetin, and provide the targeted basis for clinical Atrial fibrillation treatment.

## Introduction

Atrial fibrillation (AF) is by far the most common type of atrial arrhythmia and is a risk factor for increased cardiovascular mortality^1^. The prevalence of AF increases with age, doubling every 10 years after age 50 years and reaching 10% in patients ≥ 80 years. Furthermore, the number of patients with AF is expected to increase significantly in the coming decades^2,3^. Oral anticoagulant drugs are currently one of the main treatment modalities for AF, such as rivaroxaban and dabigatran^4,5^. In addition, relevant studies have reported that AF patients will increase significantly in the next few decades. Therefore, exploring new drugs to reduce the danger of bleeding in patients with AF, improve the cardiac function of patients and the complications of AF requires further research.

Quercetin (Que) is widely found in vegetables and fruits, and Que is also found in several herbal medicines. The therapeutic potential of Que has been widely studied because of its ability to inhibit low-density lipoprotein (LDL) oxidation, reduce adhesion molecules and other inflammatory markers, prevent neuronal oxidation and inflammatory damage, and platelet anti-aggregation^6,7^. Que has been reported to show significant therapeutic effects in animal models of heart failure, arrhythmias and hypertension in various cardiovascular diseases^8–11^. A randomized clinical trial showed that with the increase of Que dosage, the systolic and diastolic blood pressure in hypertensive patients were significantly reduced^12^. Recent studies have found that in a rat model of arrhythmia after myocardial ischemia and reperfusion injury, the administration of Que significantly reduced the time to arrhythmia as well as lactate dehydrogenase (LDH) levels in myocardial injury^13^. Based on these comprehensive data, we speculate that Que is one of the new therapeutic approaches for the prevention of cardiovascular diseases, but there is still lack of the related research in vitro and in vivo as well as clinical studies of Que for AF treatment, so we think it is necessary to do the systematic and in-depth studies on the molecular mechanisms of Que for AF treatment.

**Figure 1:**
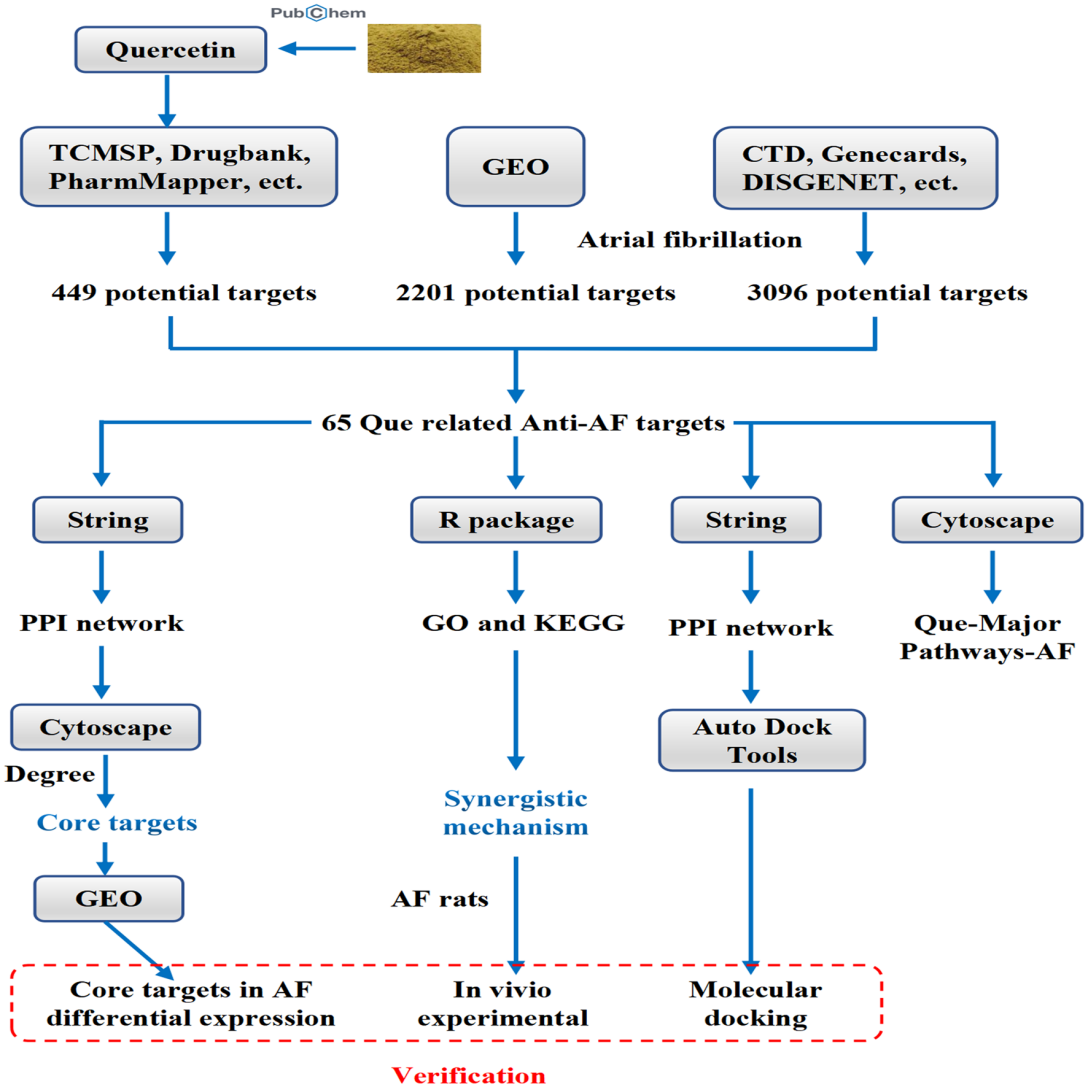
Graphical abstract.

Network pharmacology is to further analyze the relationship between drugs, diseases and targets by collecting various database data, data analysis and simulation^14^. Therefore, by using a network pharmacological analysis method, we investigated the targets of action in Que for the treatment of AF and analyzed their relevant target biological pathways, with further experiment validation in rat AF model in vivo, which laid a good foundation for further in-depth study of the mechanism of action of Que for the treatment of AF. The detail flow chart of this study is shown in Fig. 1.

## Results

### Information on potential targets of Que and AF

**Figure 2:**
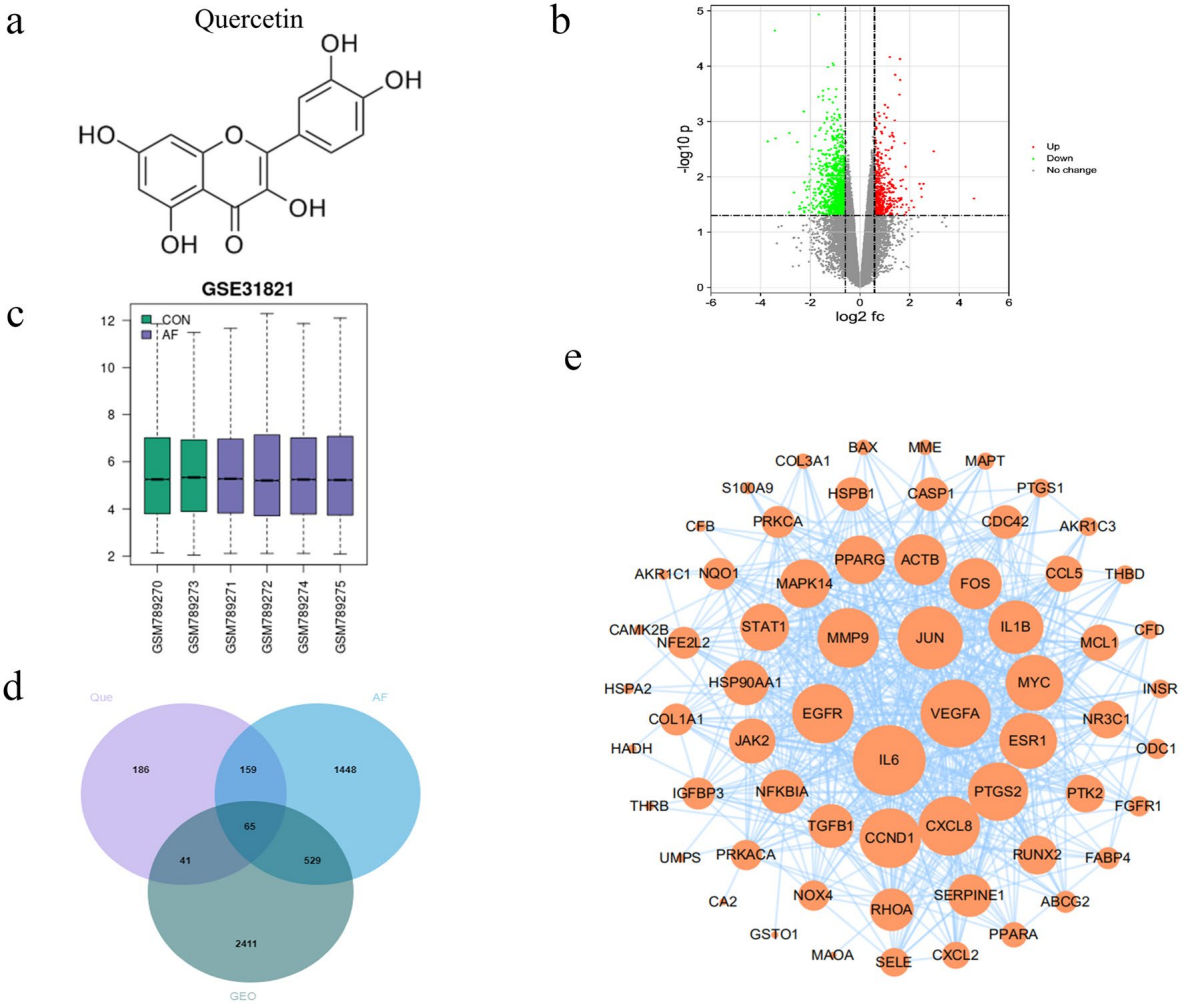
Protein–protein interaction (PPI) networks construction for target proteins of Que against AF. (**a**) The chemical structure of the quercetin. (**b**) GEO Volcano Map. Red upregulates the target, green downregulates the target; (**c**) GEO Difference Chart Differential. Gene expression in normal subjects (green) vs AF patients (purple). (**d**) Venn diagram of Que AF and GEO. (**e**) PPI network of Que against AF. The larger the circle and the darker the color, the stronger the correlation with the therapeutic target.

The chemical structure of quercetin is shown in Fig. 2a. Que potential targets were obtained by TCMSP, PharmMapper, Swiss Target Prediction, Drugbank, Stitch and Binding database databases, and a total of 449 were obtained by screening de-duplication and integration. A total of 2201 disease targets were screened using CTD, GeneCards, Disgenet and TTD databases, and 3096 differential genes between normal and AF patients were obtained using the GEO database, of which 462 were upregulated and 2634 were downregulated. From the volcano plot (Fig. 2b) and the difference plot (Fig. 2c), it can be seen that there are significant genetic differences between normal human atrial tissues and atrial tissues from AF patients. Combining drug targets, disease targets and GEO targets, 65 core targets, including IL-6, EGFR, MMP9, ESR1 (etc.) were obtained by online mapping of Venn diagram (Fig. 2d).

### PPI network and key targets of Que for AF

The Que and AF intersection targets were imported into String database, species limited to human, and the protein interactions network was obtained, which was further imported into Cytoscape for topological analysis, and the results suggested that there were 65 nodes and 575 edges in the network, and the key core targets were obtained using cytoHubba plugin (Fig. 2e), ranking the top 10 key targets for treating AF targets IL-6, VEGFA, JUN, MMP9, EGFR, CCND1, CXCL8, PTGS2, ESR1 and MYC, and the detailed information of the top ten targets ranked by the key target Degree value is shown in Table 1.

**Table 1:** Key target information table of Que in the treatment of AF.

Uniprot ID	Gene name	Protein name	Degree
P05231	IL-6	Interleukin-6	47
P15692	VEGFA	Vascular endothelial growth factor A	45
P05412	JUN	Transcription factor AP-1	41
P00533	EGFR	Epidermal growth factor receptor	38
P14780	MMP9	Mitogen-activated protein kinase 1	38
P24385	CCND1	G1/S-specific cyclin-D1	38
P10145	CXCL8	Interleukin-8	38
P35354	PTGS2	Prostaglandin G/H synthase 2	37
P03372	ESR1	Estrogen receptor	35
P01106	MYC	Myc proto-oncogene protein	35

### GO function analysis and classification

**Figure 3:**
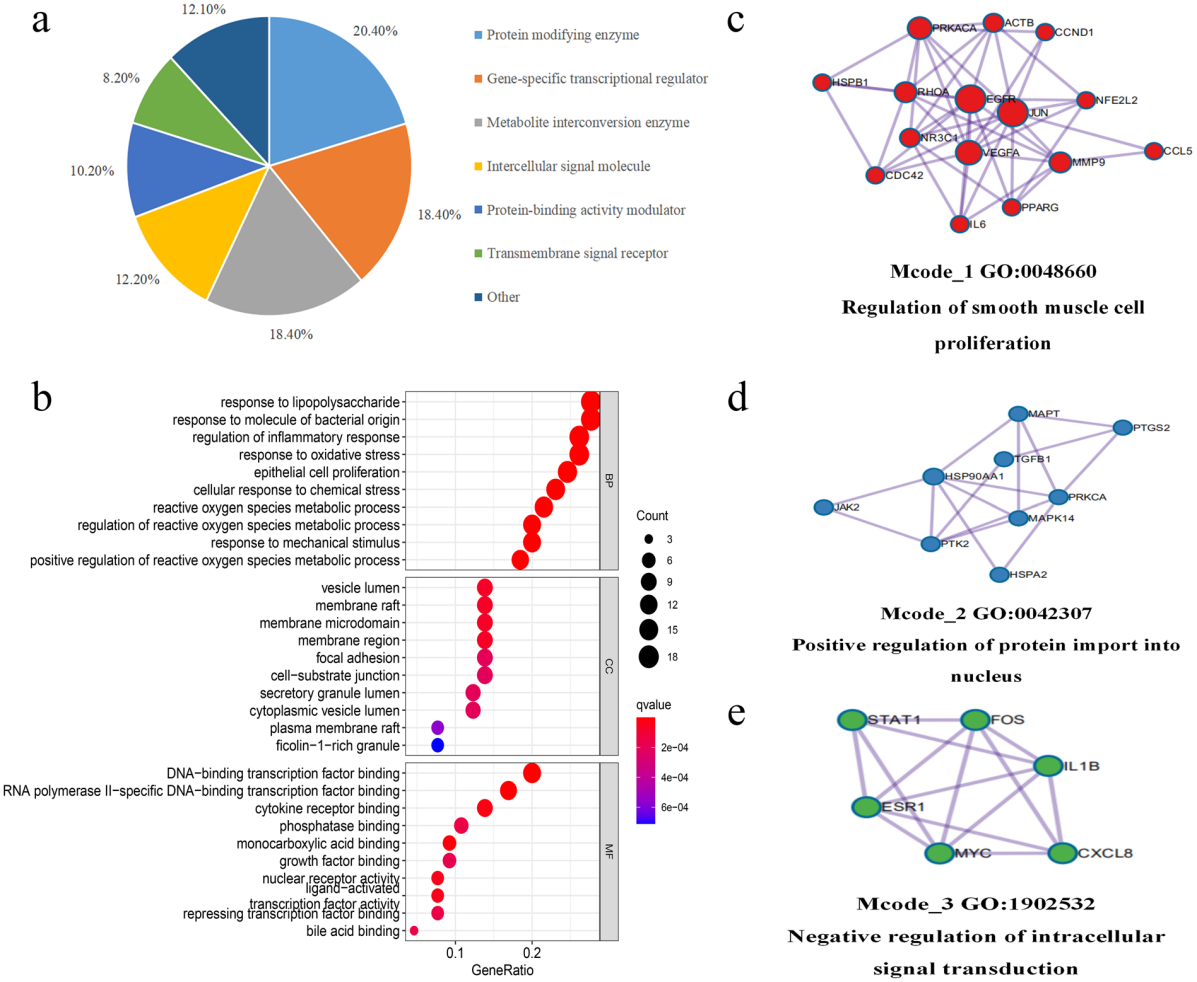
Bioinformatics analysis of target proteins of Que against AF. (**a**) Panther classification categorized target proteins of Que against AF. The figures next to the pie chart indicate the percentage of protein in the given functional class. (**b**) GO, BP, CC and MF enrichment analysis of interselection targets. The intensity of the color represents the adjusted p value, and the bubble size corresponds to the number of genes. (**c**–**e**) The Molecular Complex Detection (MCODE) algorithm has been used to identify densely connected network components.

To further clarify the mechanism of action of quercetin in the treatment of AF, we classified 65 core targets into proteins using the PANTHER database (Fig. 3a), found that they were mainly focused on protein modifying enzymes (20.4%), gene-specific transcriptional regulators (18.40%), and metabolite interconverting enzymes (18.40%). 65 core genes were introduced into the R package, and the GO functional results (Fig. 3b), the biological process (BP) was mainly focused on: response to lipopolysaccharide, response to molecule of bacterial origin, regulation of inflammatory response, response to oxidative stress, and epithelial cell proliferation; cell composition (CC) is mainly focused on: vesicle lumen, membrane raft, membrane microdomain, membrane region, focal adhesion; molecular functions (MF) mainly focus on: DNA-binding transcription factor binding, RNA polymerase II-specific DNA-binding transcription factor binding, cytokine receptor binding, monocarboxylic acid binding, and growth factor binding. In addition, we used the MCODE tool to identify highly interconnected clusters in the PPI network, and the MCODE clusters were classified into three types, including regulation of smooth muscle cell proliferation (Fig. 3c), positive regulation of protein import into nucleus (Fig. 3d), negative regulation of intracellular signal transduction (Fig. 3e).

### KEGG pathway analysis and core pathway network construction

**Figure 4:**
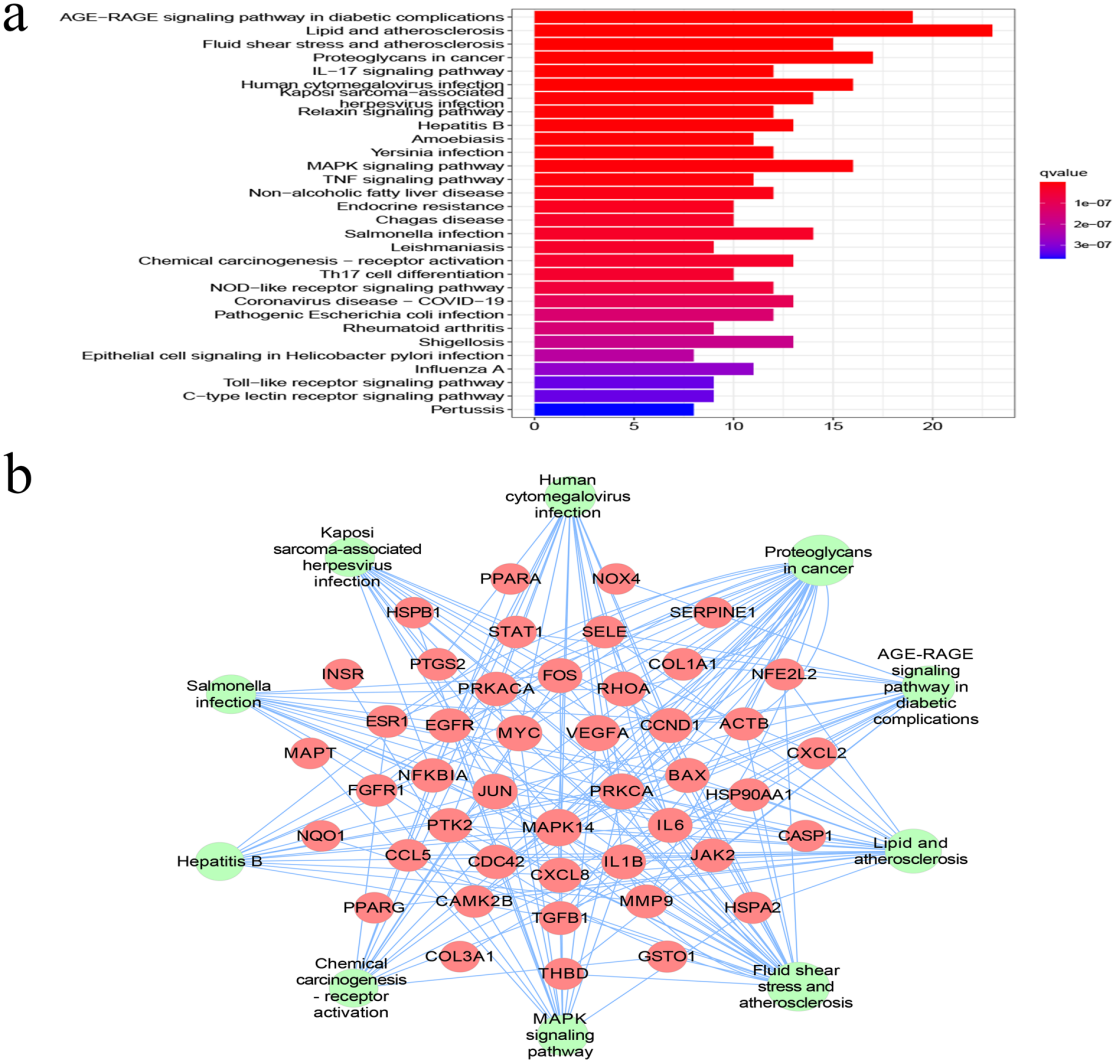
KEGG pathway enrichment analysis. (**a**) KEGG enrichment analysis of interselection targets. The intensity of the color represents the adjusted P value. (**b**) Que target-major pathway-AF. The middle purple circle is the relevant pathway and the outer circle is the relevant target in the pathway. (**c**) MAPK detailed pathway map. The red area is the MAPK upregulation target of the key pathway of AF for Que treatment.

The pathways of action were clarified by KEGG enrichment analysis of 65 intersecting genes, with 157 enrichment terms, of which the top 30 most significant pathways (Fig. 4a), the core targets may affect the lipid and atherosclerosis, AGE-RAGE signaling pathway in diabetic complications, MAPK signaling pathway, and IL-17 signaling pathway, suggesting that Que may be effective in treating AF by modulating the above signaling pathways.

**Figure 5:**
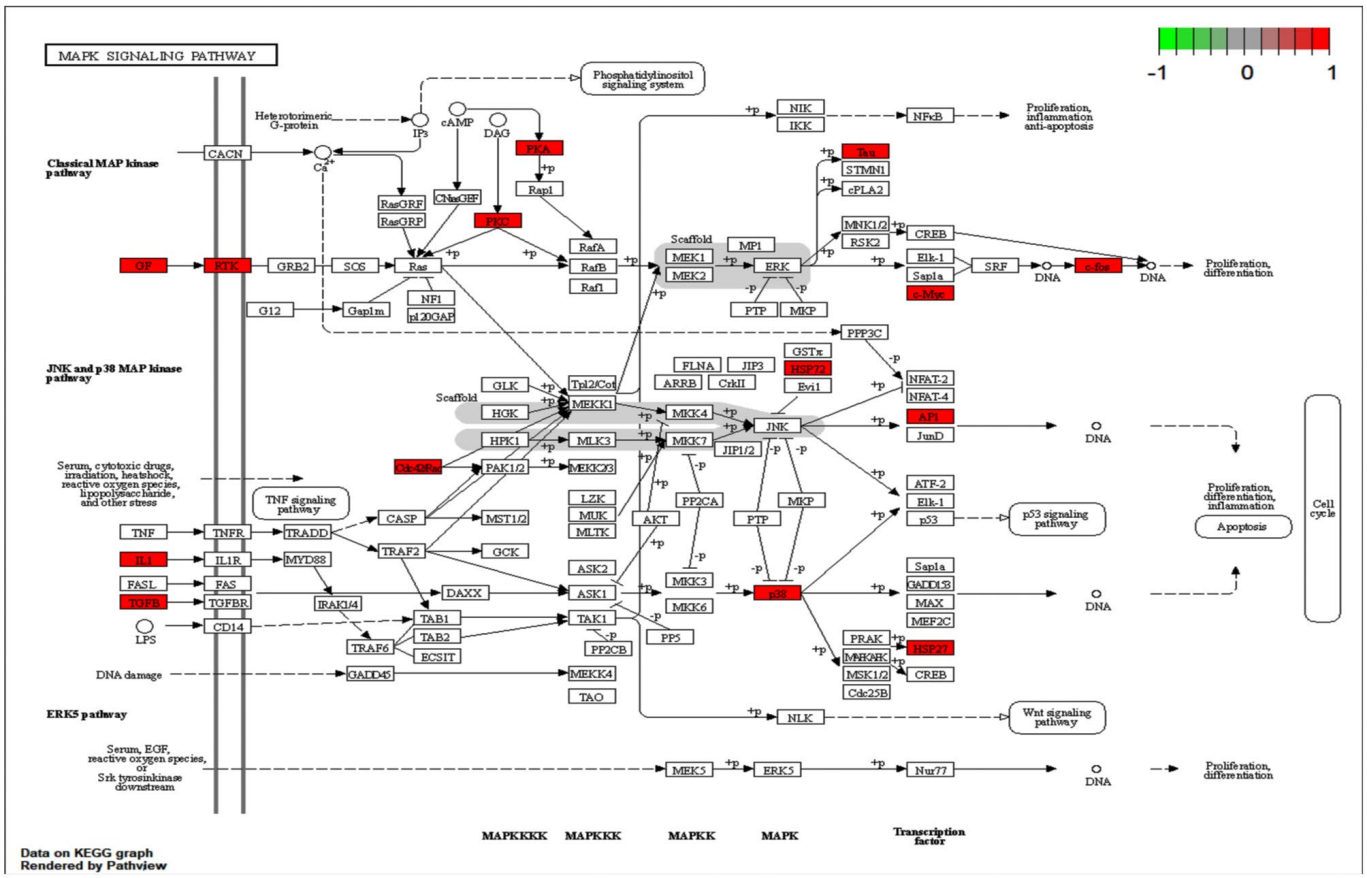
MAPK signaling pathway, the red area was signal upregulation.

The top 10 relevant pathways of Que for AF were screened (Table 2) and imported into Cytoscape to construct the “Que-Major Pathways-AF” network (Fig. 4b). After screening out the KEGG-enriched pathways that were not related to AF disease, it was found that the important pathway in Que for AF treatment was probably related to MAPK signaling pathway, and the detailed pathway map was obtained by R package analysis (Fig. 5), in which the red area was signal upregulation.

**Table 2:** KEGG pathway enrichment analysis of Que against AF.

Pathway	Enrichment	P value	Symbols	Count
AGE-RAGE signaling pathway in diabetic complications	29.23	7.01E − 22	NOX4, VEGFA, CCND1, BAX, JUN, IL-6, PRKCA, STAT1, IL-1B, SELE, CSCL8, TGFB1, THBD, SERPINE, COLA1A, COL3A1, JAK2, MAPK14, CD42	19
Lipid and atherosclerosis	35.38	1.45E − 20	PTK2, MMP9, CAMK2B, PPARG, FOS, BAX, JUN, IL6, NFKBIA, PRKCA, IL1B, SELE, CXCL8, NFE2L2, CXCL2, CASP1, JAK2, MAPK14, HSP90AA1, CCL5, RHOA, CDC42, HSPA42	23
Fluid shear stress and atherosclerosis	23.08	1.51E − 13	PTK2, MMP9, VEGFA, FOS, JUN, IL1B, SELE, THBD, NFE2L2, NQ01, MAPK14, HSP90AA1, RHOA, GSTO1, ACTB	15
Proteoglycans in cancer	26.15	2.33E − 13	PTK2, MMP9, CAMK2B, VEGFA, CND1, PRKCA, MYC, TGFB1, COL1A1, FGFR1, ESR1, PRKACA, MAPK14, RHOA, CDC42, ACTB	17
Human cytomegalovirus infection	24.61	1.41E − 11	PTK2, PTGS2, VEGFA, CCND1, BAX, IL6, NFKBIA, PRKCA, MYC, IL1B, CXCL8, PRKACA, MAPK14, CCL5, RHOA	16
Kaposi sarcoma-associated herpesvirus infection	21.54	2.64E − 10	VEGFA, CCND1, FOS, BAX, JUN, IL6, NFKBIA, STAT1, MYC, CXCL8, CXCL2, JAK2, MAPK14	14
Hepatitis B	20.00	3.34E − 10	FOS, BAX, JUN, IL6, NFKBIA, PRKCA, STAT1, MYC, CXCL8, TGFB1, JAK2, MAPK14	13
MAPK signaling pathway	24.62	7.75E − 10	MAPT, INSR, VEGFA, FOS, JUN, PRKCA, MYC, IL1B, HSPB1, TGFB1, FGFR1, PRKACA, MAPK14, CDC42, HSPA2	16
Salmonella infection	21.54	6.95E − 09	BAX, JUN, IL6, NFKBIA, MYC, IL1B, CXCL8, CASP1, MAPK14, HSP90AA1, RHOA, CDC42, ACTB	14
Chemical carcinogenesis—receptor activation	20.00	9.16E − 09	VEGFA, CCND1, FOS, JUN, PRKCA, MYC, PPARA, JAK2, ESR1, PRKACA, HSP90AA1, GSTO1	13

### Molecular docking and differential analysis of core targets

**Figure 6:**
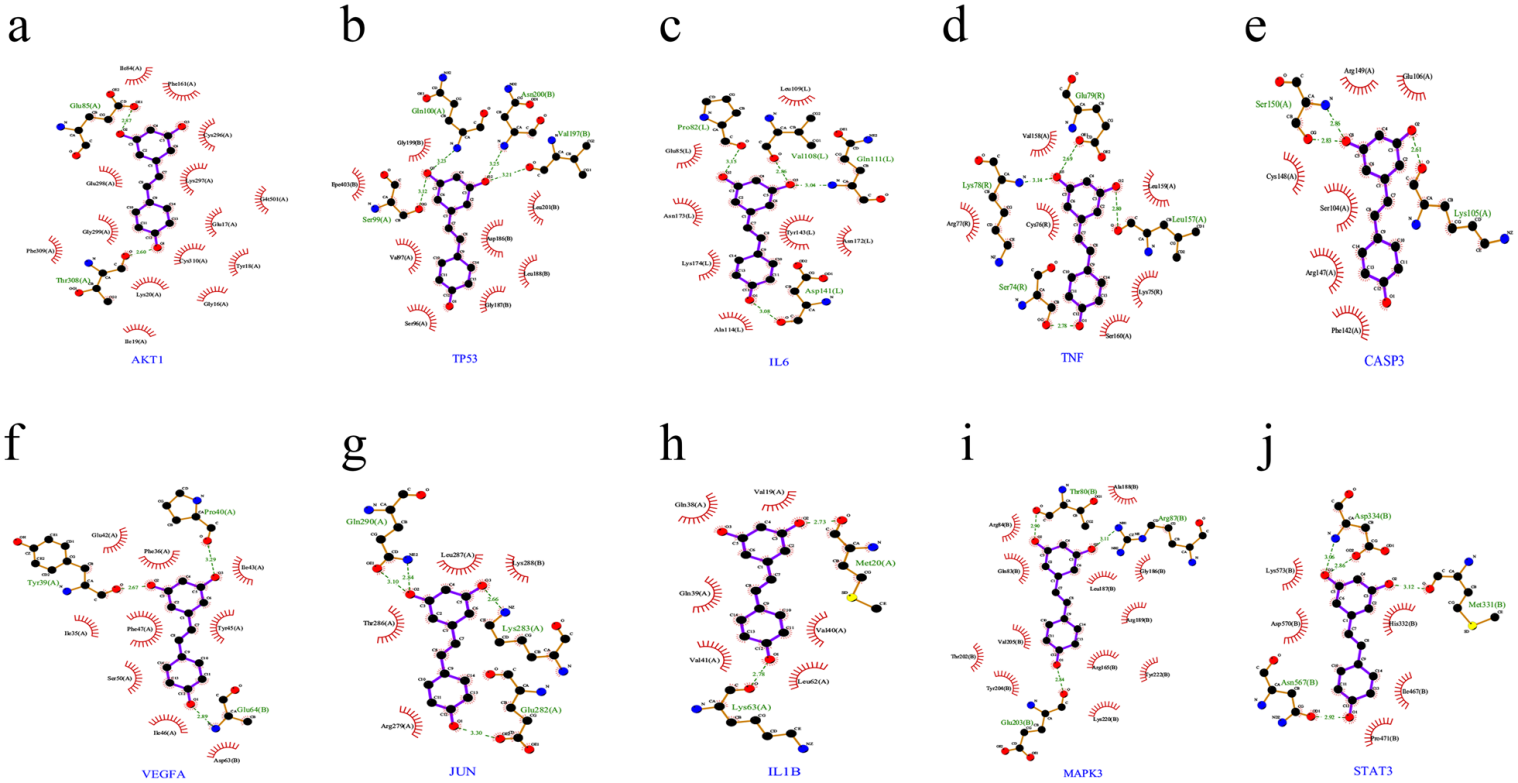
Docking pattern of Que with key target molecules. (**a**) IL-6 and Que molecular docking; (**b**) VEGFA and Que molecular docking; (**c**) JUN and Que molecular docking; (**d**) MMP9 and Que molecular docking; (**e**) EGFR and Que molecular docking.

The five core targets with high Degree values were selected for molecular docking to verify the binding activity of Que active ingredients to potential anti-AF targets. The average docking affinity of the results was − 5.042 kcal/mol, as detailed in Table 3, where the larger the absolute value of the docking affinity, the more stable the binding proved to be, indicating that Que has strong binding to the core targets. The detailed diagram of docking with each key target site is shown in Fig. 6.

**Table 3:** Molecular docking results.

Compound	Target	PDB	Energy (kcal/mol)
Quercetin	IL-6	4O9H	− 4.7
Quercetin	VEGFA	4G1W	− 6.47
Quercetin	JUN	1JUN	− 3.64
Quercetin	MMP9	5TH6	− 5.15
Quercetin	EGFR	3IKA	− 5.25

**Figure 7:**
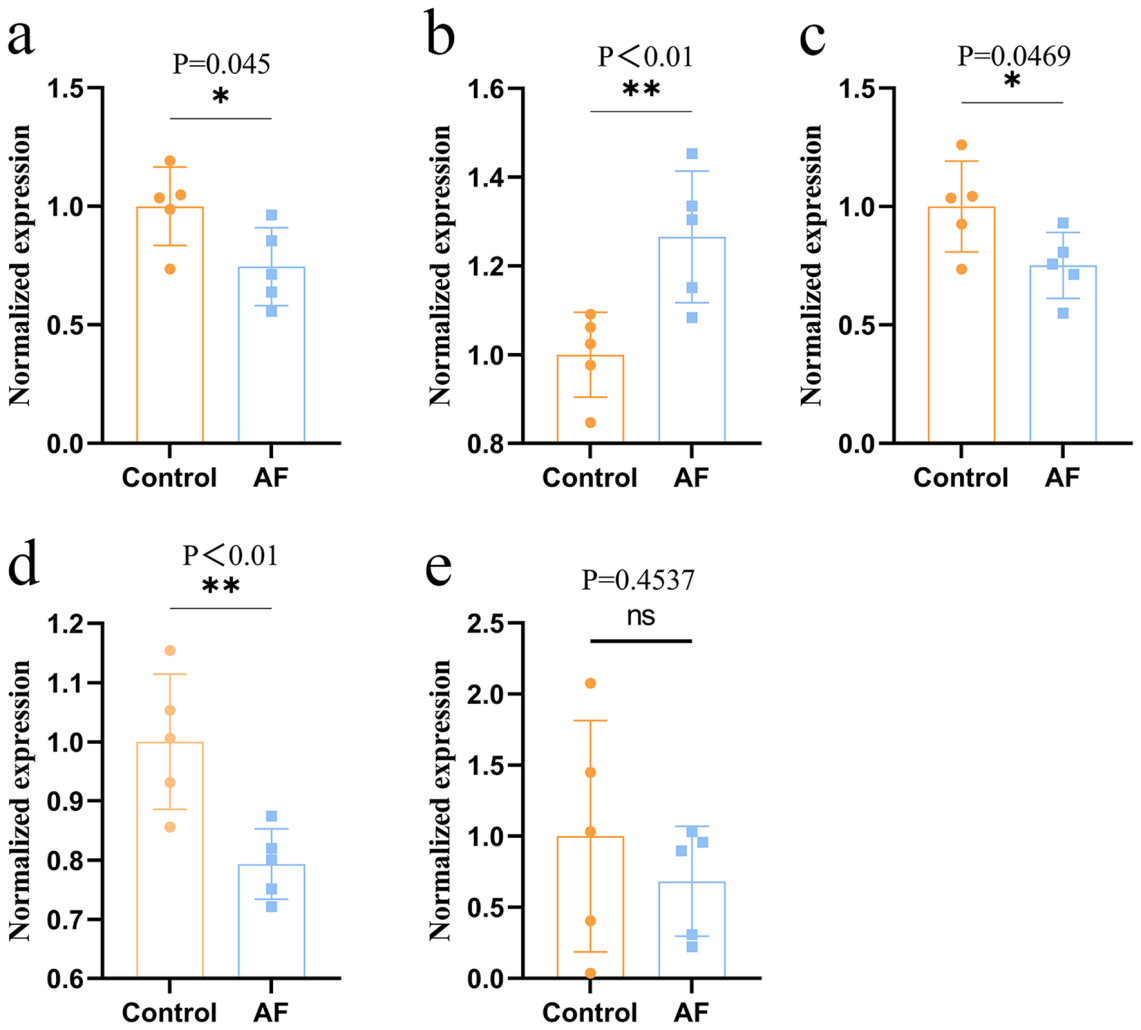
The core action targets of Que on AF were differentially expressed in the GEO data set. Left atrial healthy controls n = 5, AF group n = 5. Values are expressed as mean ± SD. Compared with Control, *P < 0.05 and **P < 0.01. (**a**) IL-6 differential expression; (**b**) VEGFA differential expression; (**c**) JUN differential expression; (**d**) EGFR differential expression; (**e**) MMP9 differential expression.

Using the difference data of control group and AF group in GEO database, the differential expression of the first five core targets in Que treatment of AF diseases (Fig. 7). VEGFA was significantly upregulated, IL-6, JUN and VEGFA were significantly downregulated compared to controls, while MMP9 targets were not statistically significant, which was considered to be related to the small sample size. The above results suggest that the core targets are closely related to the pathological changes of AF.

### Effect of quercetin on atrial fibrillation

**Figure 8:**
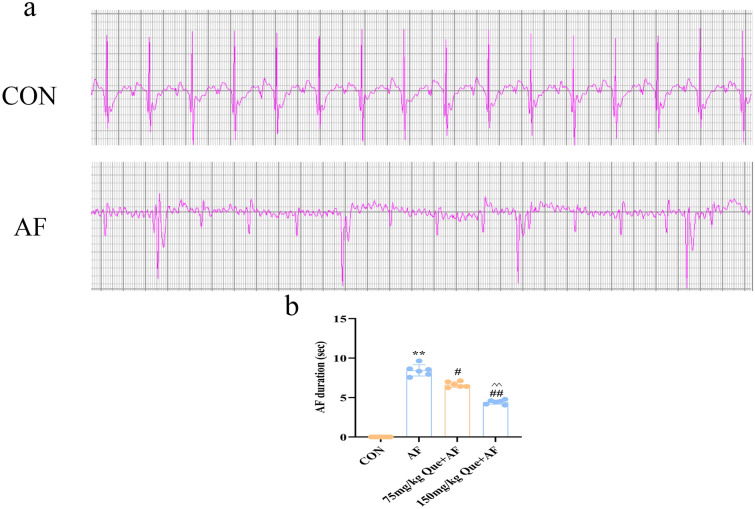
Que reduces the duration of paroxysmal AF (n = 6 for each group). (**a**) A rat model of AF was successfully established. (**b**) Duration of AF. **P < 0.01 VS CON; ^#^P < 0.05 VS AF, ^##^P < 0.01 VS AF; ^^^^P < 0.01 VS 75 mg/kg Que + AF.

In contrast with the visible of p-wave in CON group, in typical AF and Que + AF groups, f-wave was appeared of and of p-wave was disappeared by electrocardiogram measurement (Fig. 8a). Compared with AF group, the duration of AF was significantly reduced with the increasing concentrations of Que (P < 0.01) (Fig. 8b).

### Effects of quercetin on cardiac function in rats with atrial fibrillation

**Figure 9:**
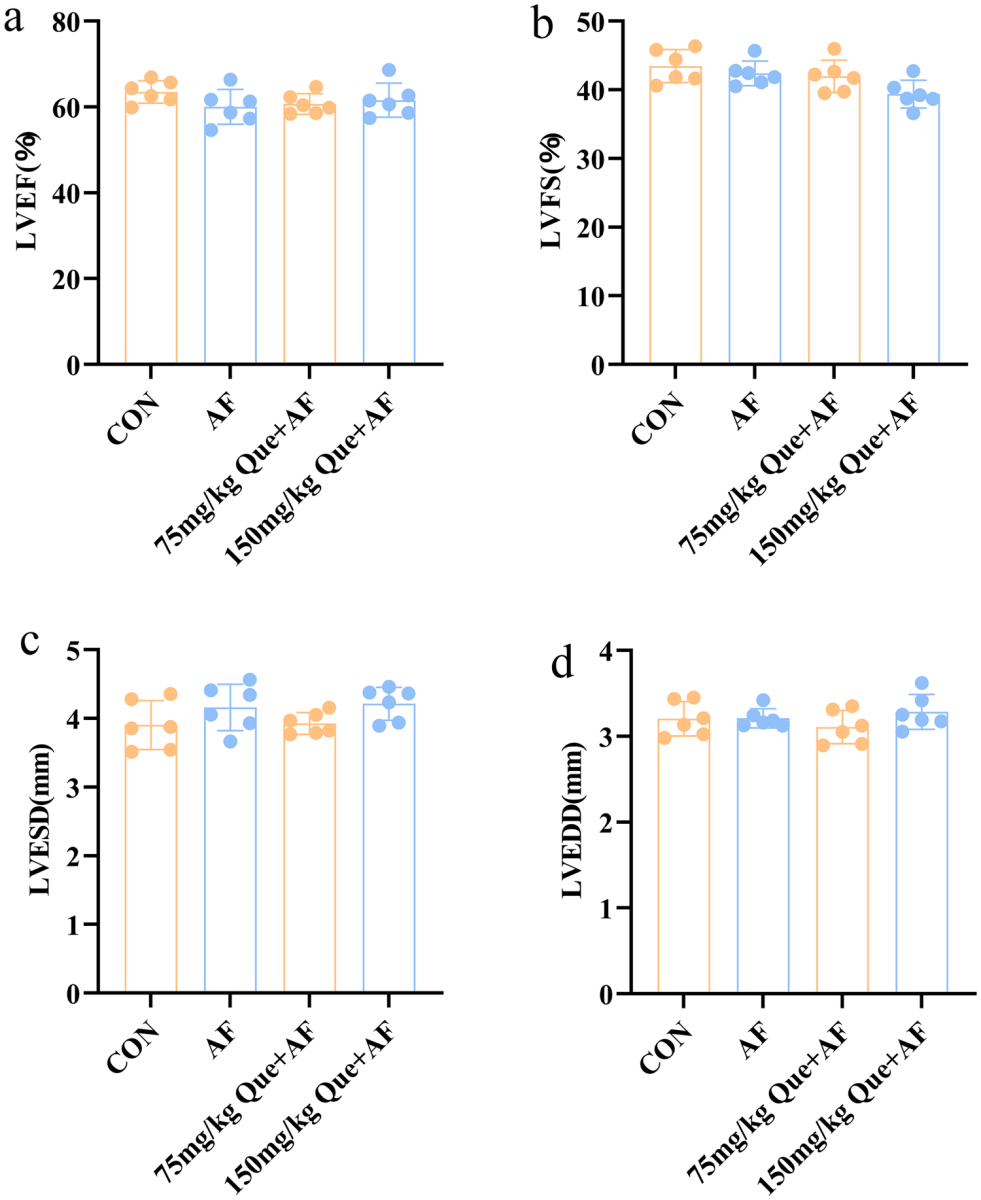
Que had no significant effect on LV function in each group (n = 6). (**a**) LVEF: left ventricular ejection fraction. (**b**) LVFS: left ventricular shortening fraction. (**c**) LVESD: left ventricular end-systolic diameter. (**d**) LVEDD: left ventricular end-diastolic ductal diameter.

No significant changes in cardiac function were happened in the CON, AF and Que + AF groups according to the cardiac ultrasound results (Fig. 9).

### Atrial tissues pathology observation

**Figure 10:**
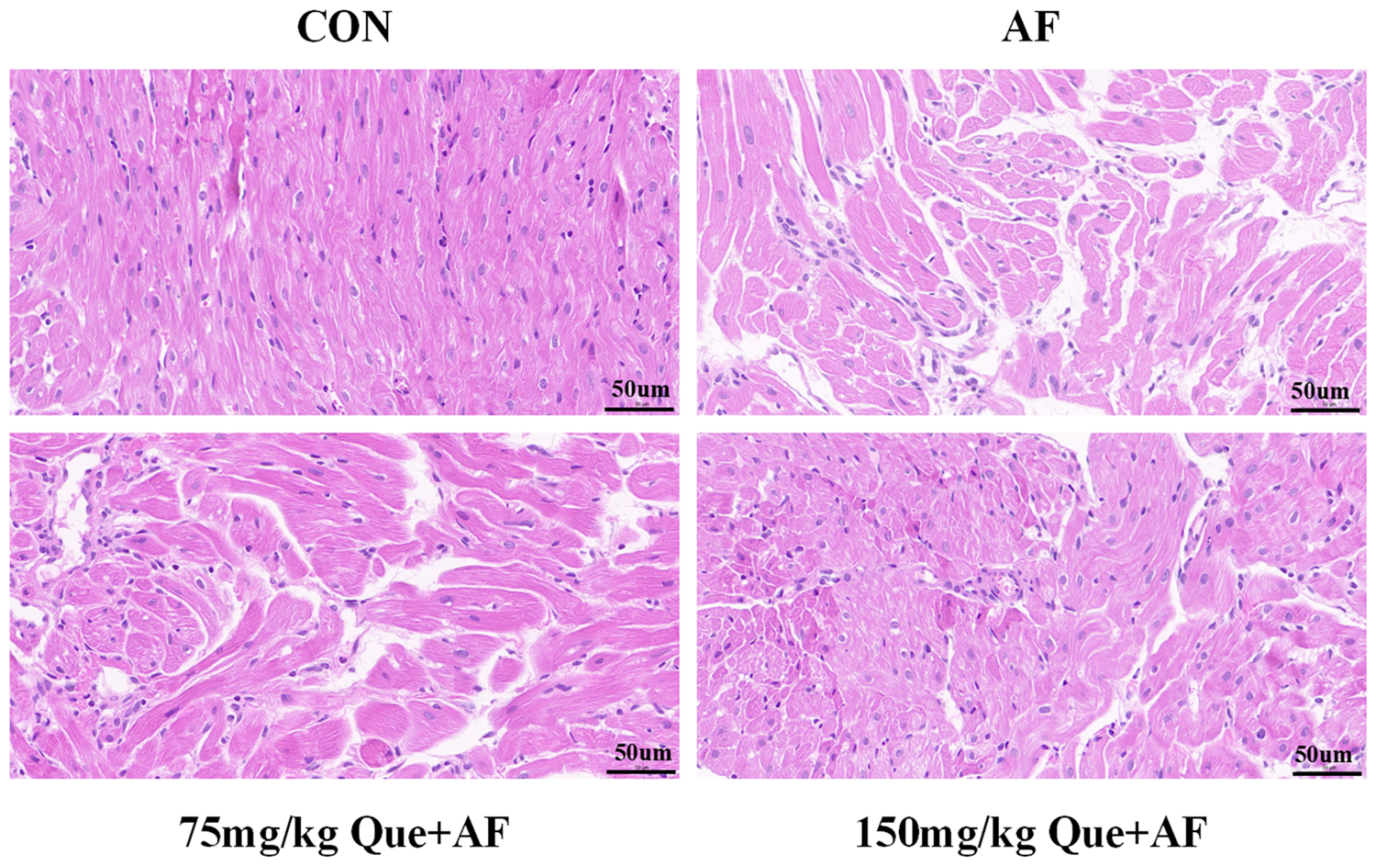
Morphological and structural changes of atrial myocytes in each group of rats.

HE staining results showed that the atrial tissues in the CON group were densely and neatly arranged, disorganized arrangement, hypertrophy and widened cell gaps of the atrial myocytes were showed in the AF group. Compared with the AF group, the morphology and the degree of structural damage of atrial myocytes were improved significantly with the increasing concentrations of Que (Fig. 10).

**Figure 11:**
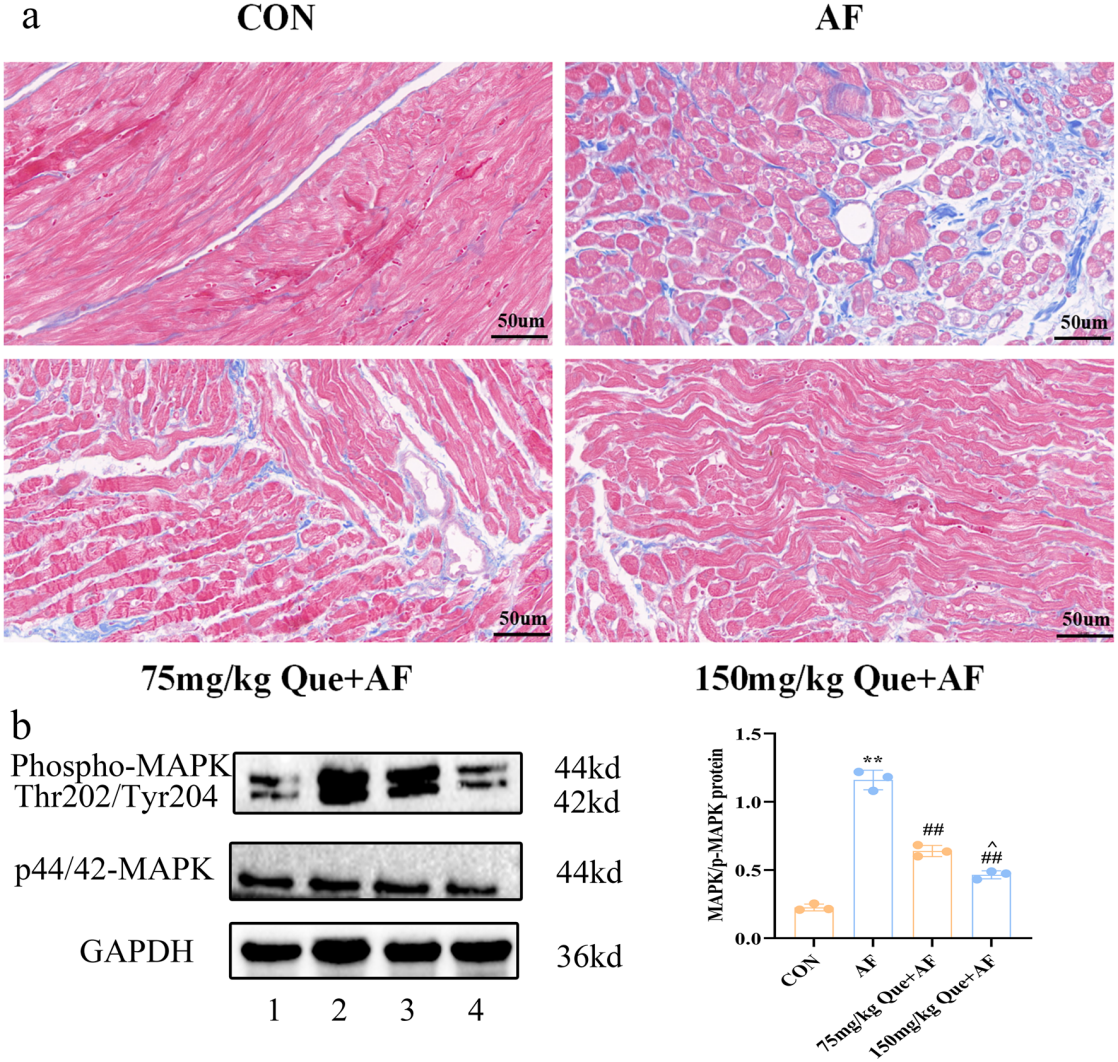
(**a**) Changes in atrial muscle fibrosis in each group. (**b**) Differences in MAPK and P-MAPK protein expression in each group. 1: CON, 2: AF, 3: 75 mg/kg Que + AF, 4: 150 mg/kg Que + AF. **P < 0.01 VS CON; ^#^P < 0.05 VS AF, ^##^P < 0.01 VS AF; ^^^P < 0.05 VS 75 mg/kg Que + AF.

The masson staining results showed that in the atrial myocardial tissue of the CON group, no significant proliferation of blue collagen fibers was happened, and the numbers of collagen fibers in atrial tissues of AF was increased. Compared with the AF group, the numbers of collagen fibers in atrial tissues were decreased significantly with the increasing concentration of Que (P < 0.05) (Fig. 11a).

### Effect of Que on protein expression in AF atrial tissue

Compared with the CON group, p-MAPK protein expression was increased in the AF group (P < 0.01). Compared with the AF group, p-MAPK protein expression was significantly decreased with the increasing concentration Que (P < 0.01). Compared with 75 mg/kg Que, 150 mg/kg Que in the AF group, p-MAPK protein expression was reduced (P < 0.05). The expression of MAPK protein in 75 mg/kg Que and 150 mg/kg Que groups were no significant difference (P > 0.05) (Fig. 11b).

## Discussion

One study found that the increased risk of cardiovascular disease in some populations was associated with low dietary intake of flavonoids, including Que^15^. It has been found that Que played the anti-ischemic and anti-arrhythmic effects in clinical studies^9–11^, it leads to great interest and concern of scientists, but the specific mechanisms of Que in the treatment of AF remains unclear. In this study, we investigated the mechanism of Que in the treatment of AF based on network pharmacology analysis. In this study, 449 Que potential targets and 2201 AF targets were obtained from each database respectively, 3096 AF differential genes were obtained from the GEO database. The intersection of the 3 was taken to obtain 65 core genes for Que treatment of AF. The above core genes were screened for the top 5 hub target genes of IL-6, VEGFA, JUN, MMP9 and EGFR by Degree value. GO analysis confirmed that Que had the potential to play an important role in the pathogenesis of AF through multiple biological pathways, and KEGG analysis obtained key pathways of Que for AF, mainly involving lipid and atherosclerosis pathways, the AGE-RAGE signaling pathwayin diabetic complications, MAPK signaling pathway and IL-17 signaling pathway, etc. Through further screening, we found that MAPK pathway plays a key role in the treatment of AF.

IL-6 plays a key role in immune, tissue regeneration and metabolism. It was shown that IL-6 in the left atrium was found to promote early atrial fibrosis through the pSTAT3/STAT3 signaling pathway by establishing postoperative AF mice, and similarly higher IL-6 concentrations were found in the pericardial drainage fluid of patients with postoperative AF in a prospective clinical study^16^. Wu reported inhibiting IL-6 inflammatory factor release reduced early atrial fibrosis and its duration in SD rats with postoperative AF^17^. Studies based on clinical follow-up investigations have found that measurement of IL-6 concentrations could provide an independent prognostic signal as well as a predictor of recurrence for mortality in AF patients^18,19^. Therefore, IL-6 has the potential to be a therapeutic target for AF. The next key gene VEGFA is a key factor in the growth process of blood vessels and endothelial cells. Its main function is to induce endothelial cell proliferation and inhibit apoptosis^20^. It is well known that AF can lead to endothelial dysfunction^21^, and Wang reported that the plasma levels of VEGFA were significantly elevated in AF patients^22^, suggesting that endothelial dysfunction may be one of the etiologies of AF, VEGFA may be a potential therapeutic target for AF.

The next gene is Transcription factor JUN, It has been shown that the transcription factor complex AP-1 plays a major role in cardiac hypertrophy, and knockout of c-jun pure mice develops in a process leading to cardiac malformations^23^, activation of c-jun impaired cell–cell communication between atrial myocytes and ultimately promotes the development of atrial arrhythmias^24,25^. Therefore, JUN has the potential to be a therapeutic target for AF. The next gene MMP9 is a matrix metalloproteinase that plays an important role in local protein hydrolysis, cell migration, apoptosis and signaling in the extracellular matrix. During AF development, overproduction of reactive oxygen species activated MAPK signaling pathway further increasing MMP9 expression^26^. Moe GW reported inhibiting MMP9 expression in canine AF model improved cardiomyocyte hypertrophy and reduced myocardial fibrosis, as well as reduced AF duration^27^. Recent clinical studies have found that the plasma MMP9 level in AF patients were higher than that in controls, which suggested MMP9 could be as a target for the treatment of AF^18,28^. The last of the first five core genes is EGFR, whose main function is to regulate cell growth, proliferation and apoptosis, and is a key regulator of cardiac organogenesis and adult heart^29^. In AF fibrotic atrial tissues, EGFR expression was increased, probably due to the cleavage by pro-fibrotic metalloproteinases during atrial remodeling^30^. The plasma level of EGFR in clinical AF patients was significantly higher than that in the control group^31^, these results suggest that EGFR plays an important role in AF. Overall, the top five core genes were found to be supported by previous studies based on network pharmacology studies.

The results of KEGG enrichment analysis suggested that the lipid and atherosclerosis pathway, AGE-RAGE signaling pathway in diabetic complications, MAPK signaling pathway and IL-17 signaling pathway played the major roles in the treatment of AF with Que. One of the main causative factors of atherosclerosis and cardiovascular disease is dyslipidemia, which is considered one of the major risk factors for AF, and several clinical studies had demonstrated that LDL and total cholesterol were negatively correlated with the occurrence of AF^32–34^. It is suggested that lipids and atherosclerosis may be involved in the pathogenesis of AF. The AGE-RAGE signaling pathway is an important link in the development of diabetic disease, and this signaling pathway directly mediates diabetic vascular calcification and vasoconstriction-diastolic dysfunction, and a meta-analysis showed that type 2 diabetes increased the risk of AF by 40%^35^. Recent studies had found that the AGE-RAGE signaling pathway was associated with the development of AF^36,37^, and that when AGE bind to RAGE, it produced inflammatory cytokines and cell adhesion molecules that further induced structural and electrical remodeling of the atria. Raposeiras-Roubín found significantly higher AGE and RAGE plasma levels in AF patients than in normal subjects, and several prospective studies have also shown that RAGE predicts future cardiovascular events and death in diabetic patients^38^. This suggesting that this pathway may mediate the progression of AF.

MAPK signaling pathway plays an important role in cell growth, proliferation and fibroblast growth. Li found that atrial structural remodeling in an AF mouse model could be attenuated by decreasing MAPK expression^39^. It has been demonstrated that activation of phosphorylated MAPK significantly increases susceptibility to AF^40^, in detail MAPK pathway (Fig. 5), red upregulated targets get inflammation and apoptosis play an important role in the development of AF disease. Inflammation is one of the important pathogenic mechanisms of AF, and there is an increasing evidence supporting the involvement of inflammation and immune response in the development and progression of AF. Many studies have found that IL-17 signaling pathway stimulates the release of pro-inflammatory cytokines, which promote myocardial fibrosis and thus induce the development of AF^41,42^. In addition, IL-17 promotes AF development in rat model with aseptic pericarditis by promoting cardiac inflammatory expression and atrial fibrosis^41,43^. Therefore, the results of the present study suggest that these four signaling pathways may be involved in the mechanism of Que treatment of AF (“Supplementary Information S1”).

GO analysis revealed the biological processes mainly focused on responses to lipopolysaccharide, regulation of inflammatory responses, phosphatidylinositol-mediated signaling, responses to oxidative stress and responses to epithelial cell proliferation related to the aforementioned pathway-mediated biological processes, which are also consistent with the aforementioned pathway-mediated biological processes. Molecular docking also suggested strong binding of Que to the above key targets and also validated the therapeutic effect of Que on AF targets at the molecular level.

In order to further validate the results of the above network pharmacology analysis, we performed rat paroxysmal AF model in vivo. Firstly, the paroxysmal AF rat model was successfully constructed by injecting ACh-CaCl_2_ mixture solution through tail vein, and we found that AF duration could be obviously reduced by the elevation concentrations of Que. Secondly, HE staining and Masson staining of atrial tissue suggested that with the increasing concentrations of Que, Que could obviously attenuate the morphological and structural damage of atrial tissues induced by paroxysmal AF, as well as reduce atrial fibrosis. Interestingly, there was no difference about the indexes of left ventricular function in each group, however, based on the results of pathological staining results, we considered the current cardiac function was still in a compensatory stage, which was in accordance with previous studies^44^. Finally, combined with the KEGG results, we selected the MAPK pathways that was more closely related to the occurrence of AF, and found that with the increasing concentrations of Que intervention, the elevation of atrial p-MAPK protein expression caused by AF of were reduced obviously.

Therefore, combining with the network pharmacology, GEO differential expression and in vivo experiments, we hypothesize that Que can medicate some key targets such as IL-6, VEGFA, JUN, MMP9, EGFR, CCND1 and other key signaling pathways such as MAPK pathway, lipid and atherosclerosis, AGE-RAGE pathway in diabetic complications and IL-17 pathway, through which Que can inhibit inflammatory expression, oxidative stress, myocardial fibrosis and alleviate AF-induced atrial remodeling and injury.

## Conclusions

In conclusion, this study has identified the core targets and key pathways of quercetin in treatment of atrial fibrillation, which through network pharmacology combined with the GEO database. In paroxysmal atrial fibrillation rat model, quercetin has a protective effect on atrial remodeling, and its mechanism may act through the MAPK signaling pathway. Our study suggests that quercetin can ameliorate the pathophysiological process of atrial fibrillation, which possibly through the synergistic interaction between multiple core targets and pathways, and MAPK is the core target and pathway. These results provide a basis for the clinical application and basic research of quercetin in treatment of atrial fibrillation.

## Methods

### Network pharmacology

Acquisition of Que targets. Using the TCMSP (http://tcmspw.com/)^45^, PharmMapper (http://www.lilab-ecust.cn/pharmmapper/)^46^, SwissTargetPrediction (http://www.swisstargetprediction.ch/)^47^, Drugbank (https://go.drugbank.com/)^48^, Binding database (http://www.bindingdb.org/bind/index.jsp)^49^, STITCH (http://stitch.embl.de/cgi/input.pl?UserId=MxcokIPgQVMy&sessionId=O32YjbzG5Gio) and other databases to obtain Que targets, default HumanProtein Targets Only (Retrieved on:2021.7.10).

Acquisition of AF related targets. The CTD (http://ctdbase.org)^50^, GeneCards (https://www.genecards.org/)^51^, DISGENET(https://www.disgenet.org/)^52^, Therapeutic Target Database (http://db.idrblab.net/ttd/)^53^, were used to “Atrial fibrillation” was used as a keyword to search for the relevant genes. The collected targets were combined and eliminated by the median algorithm to obtain disease targets. GSE31821 data were imported into the online analysis tool GEO2R for processing to obtain differentially expressed genes. The gene screening criteria were P < 0.05 and |logFC| ≥ 0.5 to obtain differentially expressed genes between normal tissues and AF, and further volcano maps of differentially expressed genes were drawn^54^.

Cross-tabulation of drugs, diseases and GEO. By using the online mapping tool (http://www.ehbio.com/test/venn/#/), intersections of drug targets, disease targets and differential genes can be obtained.

Construction of protein–protein interaction network (PPI). The intersecting genes were imported into the String database^55^ (https://string-db.org/cgi/input.pl) to construct the protein–protein interaction network (PPI), and the species selectors, exported in tsv format. The tsv was imported into Cytoscape 3.7.2 software (http://www.cytoscape.org/) for adjustment as well as to obtain key targets using the cytoHubba plugin. Simultaneous protein functional classification of the intersecting targets using the Panther database.

GO analysis and KEGG pathway and Que-primary pathway-AF network construction. To further understand the function and role in signaling pathways of the intersecting genes, the gene abbreviations (Symbol) were transformed into gene ID (EntrezID), and the results were analyzed visually by the R language package^6^, as well as the gene ontology (GO) of the above intersecting genes, GO analysis can further explain the role of the intersecting genes in molecular function (MF), biological process (BP) and cellular component (CC) of AF resistance. The Kyoto encyclopedia of genes and genomes (KEGG)^56^ enrichment analysis was used to investigate the major anti-AF signaling pathways involved in the crossover genes. The core target network of “Que-major pathway-AF” was also constructed.

Molecular Docking and differential analysis of core targets. The known key targets were molecularly docked to Que to obtain docking affinities reflecting their stability. The PDB database was used to download the core protein molecular structures and PUBCHEM to download the structures of Que. The protein and Que structures were imported into Auto Dock Tools software for processing and molecular docking, and intermolecular docking affinities were calculated and PyMol 2.4.0 was used for visualization^57,58^. Changes in core targets were analyzed using differential expression between the normal and AF groups from the GEO database. GraphPad Prism software was used for graphical visualization. Values are expressed as mean ± SD.

### In vivo experiments

#### Materials and equipment

Quercetin (Que, purity ≥ 98%, 849061-97-8, Sigma, USA), sodium pentobarbital (West Asian reagents, China), isoflurane (Kihong reagents, China), acetylcholine (ACh) and CaCl_2_ (Sigma, USA), BSA (Biofroxx, Germany), p44/42 MAPK (137f5, CST, USA), Phospho-MAPK (p-MAPK, thr202/tyr204) (28733-1-ap, Polyclonal, China), GAPDH (Biosharp, China).

#### Animals

Twenty four male (Sprague-Dawley) SD grade rats (180–220 g) were obtained from Bengbu Medical College (SCXK-2022-0001). The animal protocol conformed to the guide for the care and use of laboratory animals published by the US National Academy of Sciences and the US National Institutes of Health (NIH Publication No. 86-23, revised 1996) and the principles of laboratory animal care established by the National Institute for Medical Research. The study was conducted in accordance with the ARRIVE guidelines. The protocol was approved by the institutional animal care and use Committee of Bengbu Medical College. The rats were randomly divided into 3 groups, control group (CON, n = 6), AF group (AF, n = 6), and quercetin + AF group (Que + AF, n = 12), where the rats in quercetin group^13^ were divided into low-dose group (75 mg/kg, n = 6) and high-dose group (150 mg/kg, n = 6), which the rats were intragastric administrated with different concentrations of Que, once/day for 21 days, and the rats in CON and AF were intragastric administrated equal dose of salineion once/day for 21 days. At the end of 21 days, the rats in Que + AF and AF groups were injected with ACh-CaCl_2_ mixture (66 ug/ml ACh + 10 mg/ml CaCl^2^) in tail vein at a dose of 1 ml/kg once a day for 1 week, and equal amount of saline was injected in tail vein in CON group, and atrial tissue was taken after 7 days for next experiment.

#### ECG data acquisition

Before the experiment, rats were anesthetized using 2% pentobarbital sodium intraperitoneally, limb leads were performed using an electronic ECG machine, ECG was recorded in each group, and non-sinus rhythm was recorded and excluded. When the models of Que + AF and AF had been established, the appearance of f-wave and disappearance of p-wave were the markers for the occurrence of AF, and the disappearance of f-wave and the appearance of p-wave were the markers for the termination of AF^44^, and the time of induction and duration of AF were recorded.

#### Echocardiography for cardiac function

After ECG recording was completed, echocardiographic testing was performed. Isoflurane anesthesia maintained with small animal ultrasound color Doppler ultrasonography (VisualSonics, Canada), the left ventricular ejection fraction (LVEF), left ventricular fractional shortening (LVFS), left ventricular end-systolic diameter (LVESD), and left ventricular end-diastolic diameter (LVEDD) were measured in each group. The mean of 3 cardiac cycles were used for statistical analysis.

#### Histopathological changes in the atria

The atrial tissues of each group were partially retained for protein extraction, and the rest were fixed in 4% paraformaldehyde for 24 h. After dehydration and embedding, paraffin sections were made, and the thickness of each section was 4 μm. HE staining and MASSON staining were performed in strict accordance with the instructions of the kit, and the morphology of atrial tissues and collagen deposition in the interstitial space of cardiomyocytes were observed under the light microscope.

#### Western blotting

The proteins were extracted from the atrial tissue homogenates of each group, electrophoresed and then transferred to the PVDF membrane (cut the length of PVDF membrane according to the molecular weight of target protein), blocking at room temperature for 2 h in 5% non-fat milk powder, where Phospho-MAPK was blocked with 5% BSA for 2 h. GAPDH (1:3000), p44/42 MAPK (1:1000) and Phospho-MAPK (1:1000) antibodies were added sequentially and incubated on a shaker at 4 ℃ for 12 h. On the next day, the PVDF membranes were washed by TBS-T, and then given a secondary antibody incubation at room temperature for 2 h, the image was acquired by chemiluminescence system (VIVILBER machine), and the grayscale values of the strips were analyzed using Image J.

#### Statistical methods

Data were statistically processed using GraphPad Prism 9.0, and all data were expressed as mean ± standard deviation. One-way ANOVA was used between groups, and P < 0.05 was statistically significant.

## Supplementary Information

Supplementary Information.

## Data Availability

The datasets generated and analyzed during the current study are available from the corresponding author on reasonable request.

## References

[1] Katritsis DG (2006). Is atrial fibrillation an inflammatory disorder?. Eur. Heart J..

[2] Go AS (2001). Prevalence of diagnosed atrial fibrillation in adults: National implications for rhythm management and stroke prevention: The AnTicoagulation and Risk Factors in Atrial Fibrillation (ATRIA) Study. JAMA.

[3] Kannel WB, Wolf PA, Benjamin EJ, Levy D (1998). Prevalence, incidence, prognosis, and predisposing conditions for atrial fibrillation: Population-based estimates. Am. J. Cardiol..

[4] Writing Group, M. (2016). Heart disease and stroke statistics-2016 update: A report from the American Heart Association. Circulation.

[5] Tomaselli GF (2020). 2020 ACC expert consensus decision pathway on management of bleeding in patients on oral anticoagulants: A report of the American College of Cardiology Solution Set Oversight Committee. J. Am. Coll. Cardiol..

[6] Torres N, Martinez-Luscher J, Porte E, Yu R, Kaan Kurtural S (2021). Impacts of leaf removal and shoot thinning on cumulative daily light intensity and thermal time and their cascading effects of grapevine (*Vitis vinifera* L.) berry and wine chemistry in warm climates. Food Chem..

[7] Flores IR (2021). Bioactive compounds in tomato (*Solanum lycopersicum*) variety saladette and their relationship with soil mineral content. Food Chem..

[8] Sharma A, Parikh M, Shah H, Gandhi T (2020). Modulation of Nrf2 by quercetin in doxorubicin-treated rats. Heliyon.

[9] Patel RV (2018). Therapeutic potential of quercetin as a cardiovascular agent. Eur. J. Med. Chem..

[10] Malishevskaia IV, Ilashchuk TA, Okipniak IV (2013). Therapeutic efficacy of quercetin in patients with is ischemic heart disease with underlying metabolic syndrome. Georgian Med. News.

[11] Chekalina NI (2017). Effect of quercetin on parameters of central hemodynamics and myocardial ischemia in patients with stable coronary heart disease. Wiadomosci lekarskie (Warsaw, Poland: 1960).

[12] Edwards, R. *et al*. Quercetin reduces blood pressure in hypertensive subjects. **137**, 2405–2411. 10.1093/jn/137.11.2405 (2007). 10.1093/jn/137.11.2405 17951477

[13] Lu J, Meng Y, Wang R, Zhang R (2020). Anti-arrhythmogenic effects of quercetin postconditioning in myocardial ischemia/reperfusion injury in a rat model. J. King Saud Univ. Sci..

[14] Silverman EK (2020). Molecular networks in Network Medicine: Development and applications. Wiley Interdiscip. Rev. Syst. Biol. Med..

[15] Knekt P, Jarvinen R, Reunanen A, Maatela J (1996). Flavonoid intake and coronary mortality in Finland: A cohort study. BMJ (Clinical Research Ed.).

[16] Liu Y (2021). Mechanism of IL-6-related spontaneous atrial fibrillation after coronary artery grafting surgery: IL-6 knockout mouse study and human observation. Translat. Res..

[17] Tsioufis C (2019). Biomarkers of atrial fibrillation in hypertension. Curr. Med. Chem..

[18] Aulin J (2020). Serial measurement of interleukin-6 and risk of mortality in anticoagulated patients with atrial fibrillation: Insights from ARISTOTLE and RE-LY trials. J. Thromb. Haemostasis JTH.

[19] Aulin J (2015). Interleukin-6 and C-reactive protein and risk for death and cardiovascular events in patients with atrial fibrillation. Am. Heart J..

[20] Tammela T (2008). Blocking VEGFR-3 suppresses angiogenic sprouting and vascular network formation. Nature.

[21] Takahashi N (2001). Atrial fibrillation impairs endothelial function of forearm vessels in humans. J. Cardiac Fail..

[22] Wang K (2019). Does an imbalance in circulating vascular endothelial growth factors (VEGFs) cause atrial fibrillation in patients with valvular heart disease?. J. Thorac. Dis..

[23] Passegué E, Jochum W, Behrens A, Ricci R, Wagner EF (2002). JunB can substitute for Jun in mouse development and cell proliferation. Nat. Genet..

[24] Yan J (2013). c-Jun N-terminal kinase activation contributes to reduced connexin43 and development of atrial arrhythmias. Cardiovasc. Res..

[25] Yan J (2021). JNK2, a newly-identified SERCA2 enhancer, augments an arrhythmic [Ca(2+)](SR) leak-load relationship. Circ. Res..

[26] Liang X (2018). Reactive oxygen species mediated oxidative stress links diabetes and atrial fibrillation. Mol. Med. Rep..

[27] Moe GW (2008). Matrix metalloproteinase inhibition attenuates atrial remodeling and vulnerability to atrial fibrillation in a canine model of heart failure. J. Cardiac Fail..

[28] Lewkowicz J (2015). MMP-9 in atrial remodeling in patients with atrial fibrillation. Ann. Cardiol. Angeiol..

[29] Iwamoto R, Mekada E (2006). ErbB and HB-EGF signaling in heart development and function. Cell Struct. Funct..

[30] Munk M (2012). Hypoxia changes the expression of the epidermal growth factor (EGF) system in human hearts and cultured cardiomyocytes. PLoS ONE.

[31] Büttner P (2019). EGF (epidermal growth factor) receptor ligands in atrial fibrillation: From genomic evidence to the identification of new players. Circ. Arrhythm. Electrophysiol..

[32] Li X (2018). Lipid profile and incidence of atrial fibrillation: A prospective cohort study in China. Clin. Cardiol..

[33] Magnussen C (2017). Sex differences and similarities in atrial fibrillation epidemiology, risk factors, and mortality in community cohorts: Results from the BiomarCaRE Consortium (Biomarker for Cardiovascular Risk Assessment in Europe). Circulation.

[34] Lopez FL (2012). Blood lipid levels, lipid-lowering medications, and the incidence of atrial fibrillation: The atherosclerosis risk in communities study. Circ. Arrhythm. Electrophysiol..

[35] Huxley RR, Filion KB, Konety S, Alonso A (2011). Meta-analysis of cohort and case-control studies of type 2 diabetes mellitus and risk of atrial fibrillation. Am. J. Cardiol..

[36] Begieneman MP (2015). Atrial fibrillation coincides with the advanced glycation end product N(ε)-(carboxymethyl)lysine in the atrium. Am. J. Pathol..

[37] Raposeiras-Roubín S (2012). Evidence for a role of advanced glycation end products in atrial fibrillation. Int. J. Cardiol..

[38] Fujisawa K (2013). Circulating soluble RAGE as a predictive biomarker of cardiovascular event risk in patients with type 2 diabetes. Atherosclerosis.

[39] Li D (2001). Effects of angiotensin-converting enzyme inhibition on the development of the atrial fibrillation substrate in dogs with ventricular tachypacing-induced congestive heart failure. Circulation.

[40] Aschar-Sobbi R (2015). Increased atrial arrhythmia susceptibility induced by intense endurance exercise in mice requires TNFα. Nat. Commun..

[41] Valente AJ (2012). Interleukin-17A stimulates cardiac fibroblast proliferation and migration via negative regulation of the dual-specificity phosphatase MKP-1/DUSP-1. Cell. Signal..

[42] Onishi RM, Gaffen SL (2010). Interleukin-17 and its target genes: Mechanisms of interleukin-17 function in disease. Immunology.

[43] Yue H (2019). Comparative transcriptome analysis to elucidate the therapeutic mechanism of colchicine against atrial fibrillation. Biomed. Pharmacother..

[44] Lv, X. *et al*. βOverexpression of miR-27b-3p targeting Wnt3a regulates the signaling pathway of Wnt/-catenin and attenuates atrial fibrosis in rats with atrial fibrillation. **2019**, 5703764. 10.1155/2019/5703764(2019). 10.1155/2019/5703764 PMC6501122 31178968

[45] Ru J (2014). TCMSP: A database of systems pharmacology for drug discovery from herbal medicines. J. Cheminform..

[46] Wang X (2017). PharmMapper 2017 update: A web server for potential drug target identification with a comprehensive target pharmacophore database. Nucleic Acids Res..

[47] Daina A, Michielin O, Zoete V (2019). SwissTargetPrediction: Updated data and new features for efficient prediction of protein targets of small molecules. Nucleic Acids Res..

[48] Wishart DS (2018). DrugBank 50: A major update to the DrugBank database for 2018. Nucleic Acids Res..

[49] Gilson MK (2016). BindingDB in 2015: A public database for medicinal chemistry, computational chemistry and systems pharmacology. Nucleic Acids Res..

[50] Davis AP (2017). The Comparative Toxicogenomics Database: Update 2017. Nucleic Acids Res..

[51] Rebhan M, Chalifa-Caspi V, Prilusky J, Lancet D (1997). GeneCards: Integrating information about genes, proteins and diseases. Trends Genet. TIG.

[52] Zhang W (2018). A global transcriptional network connecting noncoding mutations to changes in tumor gene expression. Nat. Genet..

[53] Wang Y (2020). Therapeutic target database 2020: Enriched resource for facilitating research and early development of targeted therapeutics. Nucleic Acids Res..

[54] Yu S (2021). Mechanism of action of nicotiflorin from *Tricyrtis maculata* in the treatment of acute myocardial infarction: From network pharmacology to experimental pharmacology. Drug Des. Dev. Ther..

[55] Szklarczyk D (2015). STRING v10: Protein–protein interaction networks, integrated over the tree of life. Nucleic Acids Res..

[56] Kanehisa M, Furumichi M, Sato Y, Ishiguro-Watanabe M, Tanabe M (2021). KEGG: Integrating viruses and cellular organisms. Nucleic Acids Res..

[57] Morris GM (2009). AutoDock4 and AutoDockTools4: Automated docking with selective receptor flexibility. J. Comput. Chem..

[58] Seeliger D, de Groot BL (2010). Ligand docking and binding site analysis with PyMOL and Autodock/Vina. J. Comput. Aided Mol. Des..

