# Supplementary material for: Mechanisms of Quercetin against atrial fibrillation explored by network pharmacology combined with molecular docking and experimental validation
Source: Sci Rep. 2022 Jun 13;12:9777. doi: 10.1038/s41598-022-13911-w (PMC9192746; doi:10.1038/s41598-022-13911-w)
Supplement: Supplementary file 1 — Supplementary Information. [file 41598_2022_13911_MOESM1_ESM.pdf]

Mechanisms of Quercetin against atrial fibrillation explored by network pharmacology combined with molecular docking and experimental validation

Xin Tan[1], Wei Xian[1], Xiaorong Li[1], Yongfeng Chen[1], Jiayi Geng[2], Qiyi Wang[3], Qin Gao[3,4], Bi Tang[1], Hongju Wang[1,4], Pinfang Kang[1,4]

[1]Department of Cardiovascular Disease, the First Affiliated Hospital of Bengbu Medical College, Bengbu, China;

[2]Department of Psychiatry, Bengbu Medical College, Bengbu, China;

[3]Department of Physiology, Bengbu Medical College, Bengbu, China;

[4]Key Laboratory of Basic and Clinical Cardiovascular and Cerebrovascular Diseases, Bengbu Medical College, Bengbu, China.

*Correspondence to: Hongju Wang, PhD, and Pinfang Kang, MD

Department of Cardiovascular Disease, the First Affiliated Hospital of Bengbu Medical College, 287 Chang Huai Road, Bengbu, Anhui 233004, P. R. China; Email: docwhj1101@163.com (Hongju Wang); kangpinfang.1016@163.com (Pinfang Kang)

SUPPLEMENTARY MATERIAL INVENTORY

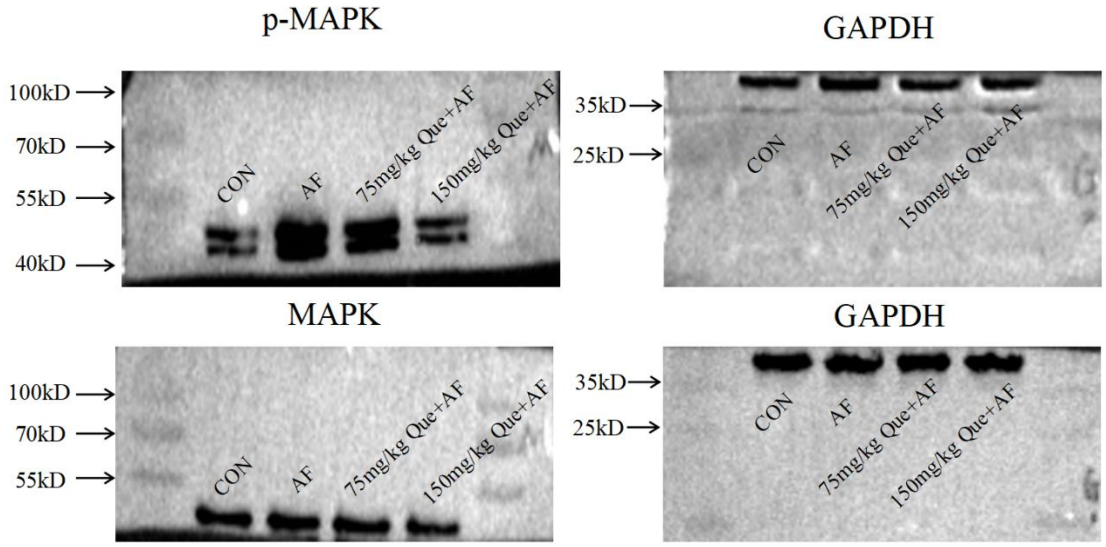

Supplementary Figure-11b. Full-length western blots for Figures 11b.

(cut the length of PVDF membrane according to the molecular weight of target protein)
